# A risk score system to timely manage treatment in Crohn’s disease: a cohort study

**DOI:** 10.1186/s12876-018-0889-5

**Published:** 2018-11-06

**Authors:** Nadia Pallotta, Giuseppina Vincoli, Patrizio Pezzotti, Maurizio Giovannone, Alessandro Gigliozzi, Danilo Badiali, Piero Vernia, Enrico Stefano Corazziari

**Affiliations:** 1 Dipartimento di Medicina Interna e Specialità Mediche, Università “Sapienza”, Policlinico “Umberto I”, V.le del Policlinico, 155, 00161 Rome, Italy; 2 Dipartimento di Malattie Infettive, Istituto Superiore di Sanità, Rome, Italy; 3 UOC di Gastroenterologia, Ospedale San Camillo De Lellis, Rieti, Italy

**Keywords:** Crohn’s disease, Risk score system, Risk factors, small intestine contrast ultrasonography, Therapy, medical, surgical

## Abstract

**Background:**

Clinical severity and intestinal lesions of Crohn’s disease (CD) usually progress over time and require a step up adjustment of the therapy either to prevent or to treat complications. The aim of the study was to  develop a simple risk scoring system to assess in individual CD patients the risk of disease progression and the need for more intensive treatment and monitoring.

**Methods:**

Prospective cohort study (January 2002–September 2014) including 160 CD patients (93 female, median age 31 years; disease behavior (B)1 25%, B2 55.6%, B3 19.4%; location (L)1 61%, L3 31.9%, L2 6%; L4 0.6%; perianal disease 28.8%) seen at 6–12-month interval. Median follow-up 7.9 years (IQR: 4.3–10.5 years). Poisson models were used to evaluate predictors, at each clinical assessment, of having the following outcomes at the subsequent clinical assessment a) use of steroids; b) start of azathioprine; c) start of anti-TNF-α drugs; d) need of surgery. For each outcome 32 variables, including demographic and clinical characteristics of patients and assessment of CD intestinal lesions and complications, were evaluated as potential predictors. The predictors included in the model were chosen by a backward selection. Risk scores were calculated taking for each predictor the integer part of the Poisson model parameter.

**Results:**

Considering 1464 clinical assessments 12 independent risk factors were identified, CD lesions, age at diagnosis < 40 years, stricturing behavior (B2), specific intestinal symptoms, female gender, BMI < 21, CDAI> 50, presence of inflammatory markers, no previous surgery or presence of termino-terminal anastomosis, current use of corticosteroid, no corticosteroid at first flare-up. Six of these predicted steroids use (score 0–9), three to start azathioprine (score 0–4); three to start anti-TNF-α drugs (score 0–4); six need of surgery (score 0–11). The predicted percentage risk to be treated with surgery within one year since the referral assessment varied from 1 to 28%; with azathioprine from 3 to 13%; with anti-TNF-α drugs from 2 to 15%.

**Conclusions:**

These scores may provide a useful clinical tool for clinicians in the prognostic assessment and treatment adjustment of Crohn’s disease in any individual patient.

## Background

Crohn’s disease (CD) has a chronic course often characterized by progression toward increasing clinical severity. The outcome of treatments for Crohn’s disease, including surgery, cannot be easily predicted since the available therapies achieve at best clinical and endoscopic remission (mucosal healing), without affecting the progressive course of the disease [1]. In addition, the course of CD varies considerably among patients making individual patient progression towards a complicated/disabling disease unpredictable. This has clinical management implications since complicated/disabling disease requires more intensive monitoring or treatment, including surgery.

Several independent predictors for complicated and severe CD have been so far identified across retrospective studies [2–4]. However, those predictors are diverse and not always consistent. Prospective studies assessing a risk prediction model are lacking**.** Although numerous techniques have been used to objectively describe disease activity and intestinal damage, no study has prospectively assessed the time-related change in severity of CD lesions nor the relative association of severity and progression of the disease. Small intestine contrast ultrasonography (SICUS) is a validated, standardized, radiation-free technique for assessment of small bowel CD lesions and associated complications [5–9]. The small amounts of oral contrast used and its simplicity make SICUS highly acceptable to patients, and well suited both for the follow-up of CD lesions and for the detection of complications.

## Methods

### Aim

The objective of this study was to develop a simple risk scoring system in order to quantify in individual patients the risk of disease progression and, among rapidly progressing CD patients, the indication for more intensive monitoring and for introducing changes in the treatment regimen in accordance with ECCO statements [10].

### Study design

We prospectively monitored all CD patients referring to two tertiary GI centers (university hospital Policlinico Umberto I, Rome and San Camillo de Lellis’s hospital, Rieti), from January 2002 to September 2014. Enrolled patients included both CD patients diagnosed prior to the study period and newly diagnosed patients in the participating hospitals during the study period.

The diagnosis of CD was based on Lennard-Jones criteria [9]. The disease characteristics at diagnosis were based on the Vienna and Montreal classifications [11]. Patients diagnosed and enrolled before 2005 were reclassified according to the Montreal classification.

All diagnosed patients then underwent complete clinical assessments at regular time intervals (6–12 months) that included both a physical examination and a clinical interview conducted by certified and experienced gastroenterologists (MG, AG, DB, PV, ESC) using a standardized clinical questionnaire. The following information was collected: symptoms, associated diseases, smoking status and load of cigarettes, family history, presence and number of surgical procedures, current treatment, BMI and Crohn’s disease activity index (CDAI), endoscopic, radiological and imaging examinations. During each clinical assessment, laboratory exams and SICUS were also routinely performed. The following detailed records of aminosalicylate (sulphasalazine or mesalazine, 2–3 g daily), corticosteroid, and immunomodulatory therapies, were collected and included: timing of administration, dose, side-effects, end and/or change of prescription. Among patients who underwent intestinal resection, the type of anastomosis as well as the pathological findings of resection specimens and of section margins, were accurately reported.

Based on the results of the clinical assessments, the participating gastroenterologists modified the patients’ treatments in accordance with the ECCO guidelines [10] using a step-up modality according to disease severity or complications. The need of additional investigations and the decision about surgery or start/change of a specific treatment class were independently decided by clinicians on clinical grounds.

Written informed consent was obtained from each subject and the study protocol was approved by the local committee of the Department of Clinical Science, University Hospital (Policlinico “Umberto I” viale del Policlinico 155, 00161 Roma, 6 December 2001).

### Small intestine contrast US

SICUS was performed using Toshiba Tosbee (Tokyo, Japan) equipment with a 3.5 MHz convex and a 5 MHz linear array transducers. The apparatus can detect a bowel wall thickness variation of 0.1 mm. All SICUS examinations were performed by an experienced sonologist (NP), who had performed more than 10,000 SICUS examinations prior to the study. SICUS was performed after overnight fasting according to a previously published method [6] and CD lesions and complications were assessed in accordance with previously published studies [5–8, 12–14]. At the end of the US investigation, we reported on a standardized form: 1) the length of any intestinal lesion as the average of at least 3 measurements; the presence of, 2) stenoses, 3) fistulas, 4) abscesses, 5) mesenteric fat hypertrophy, 6) enlarged lymph nodes and spleen, 7) colonic-ileal reflux defined as the back flow of intestinal contents from colon to ileum through the ileo-cecal valve or the ileo-colonic anastomosis (ICA).

### Statistical analysis

We developed a patient based model that considered all clinical assessments conducted and compared couples of consecutive clinical assessments on the basis of four outcomes: a) use of steroids; b) start of azathioprine; c) start of anti-TNF-α drugs; d) need of surgery. For each couple of clinical assessments, we defined the earlier visit as “referral visit”.

Thirty-two baseline variables were evaluated as potential predictors for each of the aforementioned outcomes: gender, age at diagnosis (categorized as < 20 yrs., 20 yrs. to < 30 yrs., 30 yrs. to < 40 yrs., and ≥ 40 yrs), age at study enrollment, duration of the disease, location of the disease at diagnosis [small bowel only, i.e. ileal (L1) and isolated upper disease (L4), small bowel and colon (L3), colon only (L2)], disease behavior at diagnosis (B1 non-stricturing-non-penetrating, B2 stricturing, B3 penetrating), extraintestinal manifestations, presence/absence of perianal disease at diagnosis and at each clinical assessments, steroids required for the treatment of the first flare-up, smoking habits (at diagnosis and at each clinical assessments); family history for IBD, use of steroids, use of azathioprine, use of anti-TNF-α drugs, previous surgeries, extension of resected intestine, type of ileo-colonic anastomosis [i.e latero-lateral (L-L), termino-lateral (T-L), termino-terminal (T-T)], presence of specific intestinal symptoms, serological inflammatory markers (ESR, CRP), CDAI (< 50, 50–99, ≥100), body mass index (BMI) (< 22, 21–23, 23–25, > 25), and the following SICUS findings: site and extension of CD small bowel lesions, presence of stenosis, fistulas, abscesses, enlarged lymph nodes and spleen, mesenteric fat hypertrophy, presence of colon-ileal reflux.

The baseline variables “use of steroids”, “use of azathioprine” and “use of anti-TNF-α drugs” were not assessed as potential predictors when “use of steroids”, “start of azathioprine” and “start of anti-TNF-α drugs” were identified as outcomes. Descriptive analyses were initially performed to evaluate whether some of the aforementioned predictors needed to be grouped because of low frequency and/or because they consistently occurred together. Therefore, all patients that had undergone intestinal resection were classified combining type of ileo-colonic anastomosis and extension of intestine resected as follows: L-L/T-L and intestinal resection < 20 cm; T-T and intestinal resection < 20 cm; L-L/T-L and intestinal resection ≥20 cm; TT and intestinal resection ≥20 cm. Based on the presence/absence of CD lesions and CD complications at SICUS, the following five groups of patients were identified: 1) those without CD intestinal lesion or extending ≤0.5 cm and absence of CD complications; 2) those with CD intestinal lesion extension > 0.5 cm and < 20 cm and absence of CD complications; 3) those with CD intestinal lesion extension > 0.5 cm and < 20 cm and presence of at least one CD complication; 4) those with CD intestinal lesion extension ≥20 cm and absence of CD complications; 5) those with CD intestinal lesion extension ≥20 cm and presence of at least one CD complications.

Separate multiple Poisson models were used to evaluate the potential predictors of having, within the next clinical assessment, one of the previously described outcomes. For each couple of clinical assessments in the model, time between visits was considered as exposure time. For each outcome, the predictors included in the final model were selected using a backward selection excluding at each step the variable with the highest *p*-value > 0.15 (from log-likelihood ratio test) until only variables with an adjusted p-value < 0.15 remained. In each final model for the considered outcomes selected as described above, we further grouped some categories for the following predictors: age at diagnosis, CDAI (i.e., < 50, ≥50), BMI (i.e., < 21, 21–25, > 25), SICUS findings and surgical characteristics. This was done based on the similarities of the estimated parameters on the original categories. The standard errors of the multiple Poisson parameter models were adjusted for clinical assessment clustering within the same patient. Based on the final model for each outcome, risk scores were calculated taking for each predictor the integer part of each multiple Poisson model parameter [15] which is directly related to the probability of having the outcome within the next clinical assessment. The final score for each patient at each clinical assessment is the sum of each risk factor level. The probability of having any considered outcome was calculated for each month, and up to 12 months, after a referral visit for each score. We calculated this rate as follows: month rate = exp.(constant + score). A zero score therefore represents a patient at the lowest possible risk of having that outcome. For example, the 6-month risk of having an outcome was calculated as: [1 exp.(− 6*month rate)] and expressed as a percentage. Analogously, the 9-month risk was calculated as follows: [1 exp.(− 9*month rate)].

Model-predicted and observed outcomes (1 year after the referral visit) were then graphically compared.

## Results

**Fig. 1:**
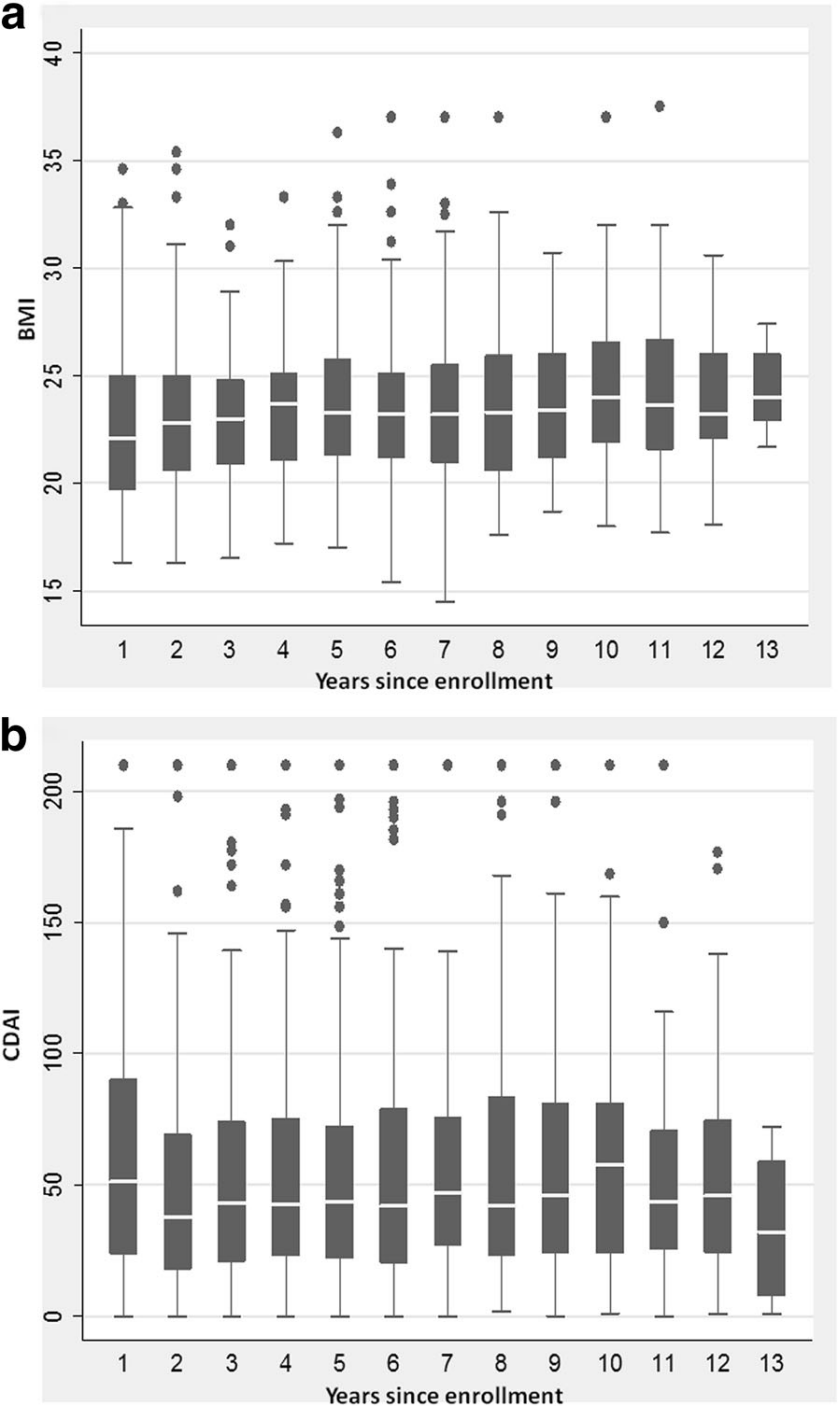
**a** Box-and-whiskers plots of BMI across 13 years follow-up period. The boxes at each score extend from the 25th percentile (× [25]) to the 75th percentile (×[75]) [i.e., the interquartile range (IQ)]; the lines inside the boxes represent the median values. The line emerging from the boxes (i.e., the “whiskers”) extend to the upper and lower adjacent values. The upper adjacent value is defined as the largest data point × [75] + 1.5 × IQ, and the lower adjacent value is defined as the smallest data point × [25] – 1.5 × IQ. Observed values more extreme than the adjacent values, if any, are individually plotted (circles). BMI (Kg/m^2^): body mass index. **b** Box-and-whiskers plots of CDAI across 13 years follow-up period. The boxes at each score extend from the 25th percentile (× [25]) to the 75th percentile (× [75]) [i.e., the interquartile range (IQ)]; the lines inside the boxes represent the median values. The line emerging from the boxes (i.e., the “whiskers”) extend to the upper and lower adjacent values. The upper adjacent value is defined as the largest data point × [75] + 1.5 × IQ, and the lower adjacent value is defined as the smallest data point × [25] – 1.5 × IQ. Observed values more extreme than the adjacent values, if any, are individually plotted (circles). CDAI: Crohn’s disease activity index

During a median follow-up period of 7.9 years (IQR: 4.3–10.5 years), 25 patients (female 11, median age 36 yrs.; disease behavior: 9 B1, 11 B2, 5 B3; disease location: 13 L1, 8 L3 2 L4, 2 L2) dropped out of study (nine moved to another town, 16 failed to present at follow-up); therefore 160 CD patients were included in the analysis for a total of 1464 assessments, being 10.5 months the minimum follow-up interval time. The main descriptive characteristics of patients at enrolment are shown in Table 1. At study enrolment, SICUS showed in 142 patients (88.8%) one or more small bowel CD lesions with a median extension of 25 cm (IQR 9–35 cm) and in 97 (60.6%) at least one CD complication. Nineteen patients (11.9%) developed complications during the follow-up period. The BMI and CDAI values measured during the study period are shown in Fig. 1. There were six deaths (two females) during the study period. Of those, three died due to renal failure and the remaining three, all on azathioprine therapy, died due to leukemia, multiple myeloma, and acute pancreatitis. After 7 years of azathioprine therapy, a female patient with terminal ileum CD, developed a Hodgkin lymphoma which was successfully treated, and subsequently a carcinoma on the ileal CD lesion.

**Table 1:** Distribution of baseline characteristics of patients at diagnosis and at inclusion

		N	%	Median	IQR
Gender	Female	93	58.1		
Age at diagnosis				31	(22.8–42.6)
Age at study enrollment				40	(29.1–53.8)
Years from diagnosis to study enrollment				6.0	(1.3–11.3)
Smoker at diagnosis	Yes	91	56.9		
Smoker at study enrollment	Yes	74	46.2		
Family history	Yes	19	11.9		
Disease Behavior	B1	40	25.0		
	B2	89	55.6		
	B3	31	19.4		
Disease Location	L1	98	61.3		
	L2	10	6.3		
	L3	51	31.9		
	L4	1	0.6		
Steroids at first flare-up	Yes	50	31.2		
Perianal Disease at diagnosis	Yes	46	28.8		
Perianal Disease at study enrollment	Yes	15	9.4		
Previous surgery	Yes	53	33.1		
Number of surgery at study enrollment	1	34			
	≥ 2	19			
Extension of intestine resected ^a^ (cm)				30	(20–40)
Type of anastomosis^a^	L-L	28	52.8		
	T-L	11	20.8		
	T-T	10	18.9		
	Stoma	1	1.9		
Abdominal symptoms	Yes	71	44.4		
Serological inflammatory markers	Yes	94	58.8		
Extra-intestinal disease	Yes	27	16.9		
Use of azathioprine	Yes	29	18.1		
Use of corticosteroids at first flare-up	Yes	50	31.2		
Use of anti-TNF- α drugs	Yes	15	9.4		
SICUS FINDINGS					
• Site of CD lesion	No lesions	18	11.2		
	Ileal	142	88.8		
	ICA	0	0.0		
• Intestinal wall thickness (mm)				8	(5–10)
• Extension of CD lesion (cm)				25	(9–35)
• Presence of strictures	Yes	83	51.9		
• Presence of fistulas	Yes	29	18.1		
• Presence of abscesses	Yes	12	7.5		
• Presence of MFH	Yes	38	23.8		
• Presence of enlarged nodes	Yes	14	8.8		
• Presence of enlarged spleen	Yes	34	21.3		
BMI				22	(19.6–25.3)
CDAI				54	(24–96)

*IQR*: Interquartile range (i.e., the first value represents the 25th percentile and the 2nd one the 75th percentile of the distribution); ^a^53 patients had a surgery before study enrollment and 50 among them had an intestinal resection

### Factors predictive of the need to use corticosteroids

**Fig. 2:**
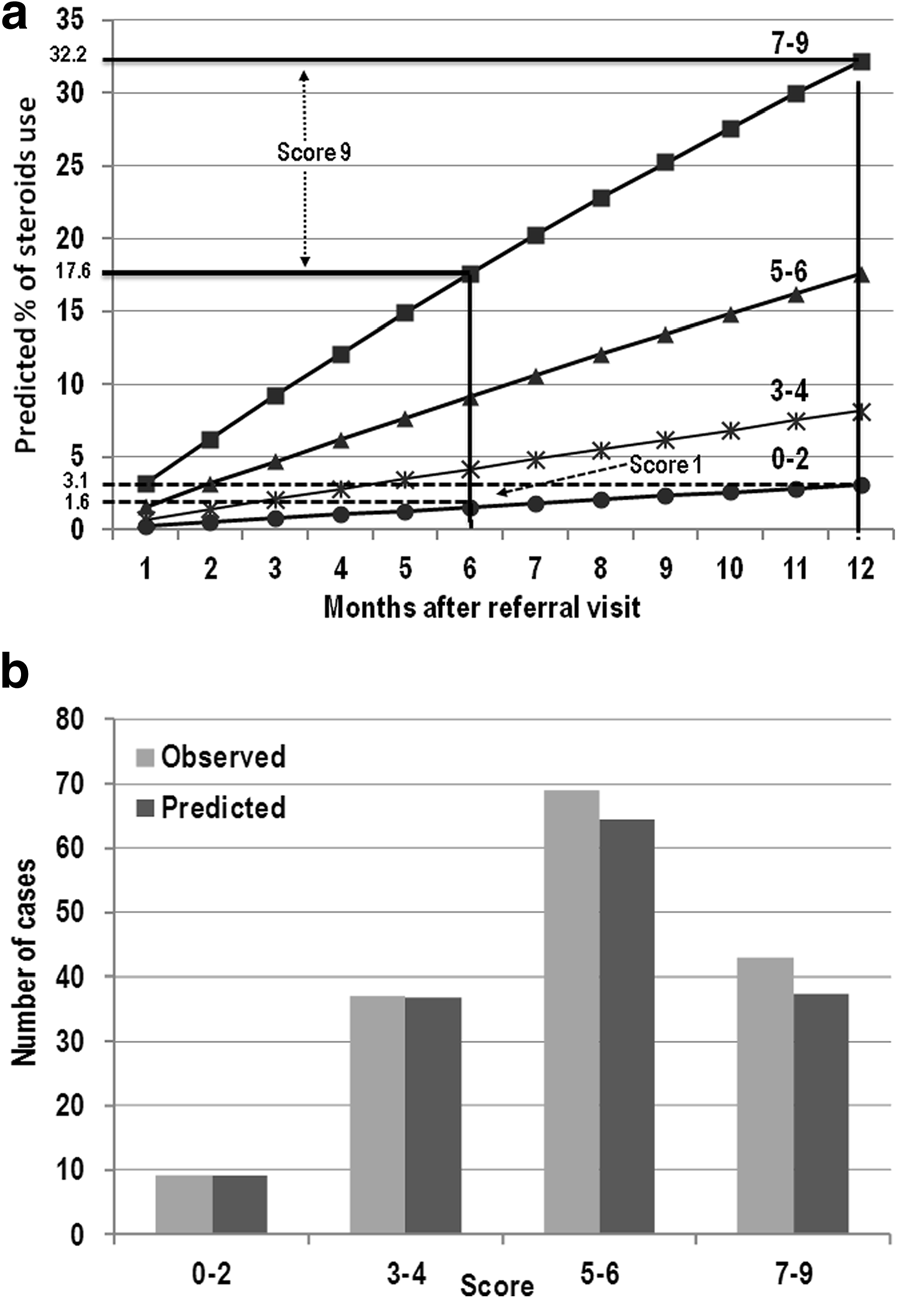
**a** Estimated cumulative probability of using corticosteroids by month after referral visit for patients with different total score. A patient scoring 1 (e.g., CD complications at SICUS, age at diagnosis ≥40 years, B1 behavior, absence of specific symptoms, presence of colon-ileal reflux at SICUS) has 1.6% and 3.1% probability of using corticosteroids at 6 months and at 1 year since the referral visit, respectively; a patient scoring 9 (e.g., CD intestinal lesions > 20 cm and no complications at SICUS, age at diagnosis < 40 years, B2 behavior, presence of specific symptoms, absence of colon-ileal reflux at SICUS) has 17.6% and 32.2% probability of using corticosteroids at 6 months and at 1 year since the referral visit, respectively. Dotted line: score 1; continuous line: score 9. **b** Observed vs model-predicted at one year since the referral visit of using corticosteroids by groups of score

Eighty nine patients (55.6%) (61 female, median age at diagnosis 30.1 years, IQR 21.2–38.1) required at least one cycle of corticosteroids. Six independent risk factors for the need of corticosteroid treatment within the next clinical assessment were identified (Table 2, section A), at SICUS evaluation the presence of 1) CD complications, 2) small bowel CD lesion > 20 cm in absence of CD complications, 3) the absence of colonic-ileal reflux; 4) age at diagnosis < 40 years; 5) stricturing (B2) behavior; 6) presence of specific intestinal symptoms. The integer risk score ranged from 0 to 9 points and observations were grouped according to the following scoring categories, 0–2, 3–4, 5–6 and 7–9. Figure 2a shows the predicted percentage risk of corticosteroids use up to 12 months after the referral visit for patients within different score groups. Figure 2b compares observed and model-predicted corticosteroid-use across the four risk groups according to the goodness-of-fit model.

**Table 2:** Adjusted incidence rate ratio (IRR) and score contribution of (A) use of corticosteroids, (B) start of azathioprine, (C) start of anti-TNF- α drugs, (D) need of surgery

	IRR	95% CI		p	score
A. Use of corticosteroids					
No or CD intestinal lesions < 20 cm and no complications (ref)	1				0
CD complications at SICUS	1.86	1.04	3.34	0.04	1
CD intestinal lesions > 20 cm and no complications at SICUS	3.38	1.80	6.35	< 0.01	3
Presence of colon-ileal reflux at SICUS (ref)	1				0
Absence of colon-ileal reflux at SICUS	1.54	1.00	2.37	0.05	1
Age at diagnosis ≥40 years (ref)	1				0
Age at diagnosis < 40 years	1.83	1.04	3.24	0.04	1
B1 or B3 disease behavior (ref)	1				0
B2 disease behavior	2.23	1.26	3.93	0.01	2
Absence of specific symptoms at clinical assessment (ref)	1				0
Presence of specific symptoms at clinical assessment	1.93	1.36	2.73	< 0.01	2
B. Start of azathioprine					
Male (ref)	1				0
Female	2.08	1.19	3.64	0.01	1
BMI > 25 (ref)	1				0
BMI < 21	4.10	1.95	8.61	< 0.01	2
BMI 21–25	2.13	1.04	4.33	0.04	1
CDAI < 50 (ref)	1				0
CDAI ≥50	1.82	1.13	2.90	0.013	1
C. Start of anti-TNF-α drugs					
Absence of CD intestinal lesions or complications at SICUS (ref)	1				0
CD intestinal lesions > 20 cm and no complications at SICUS	5.72	1.88	17.40	< 0.01	2
CD complications at SICUS	2.34	0.81	6.73	0.12	1
Negative markers of inflammation (ref)	1				0
Positive markers of inflammation	2.63	1.45	4.78	< 0.01	1
Absence of specific symptoms at clinical assessment (ref)	1				0
Presence of specific symptoms at clinical assessment	2.52	1.46	4.34	< 0.01	1
D. Need of surgery					
No previous surgery	6.40	2.01	20.31	< 0.01	2
Previous surgery and type of ileocolonic anastomosis (ICA)					
• latero-lateral ICA (ref)	1				0
• termino-terminal ICA	3.90	0.62	24.69	0.15	2
No or CD intestinal lesions and no complications (ref)	1				0
CD intestinal lesions > 0.5 cm and complications at SICUS	10.63	3.04	37.11	< 0.01	3
Corticosteroid at first flare (ref)	1				0
No corticosteroid at first flare	2.32	1.29	4.17	0.01	1
Absence of specific symptoms at clinical assessment (ref)	1				0
Presence of specific symptoms at clinical assessment	3.75	2.01	7.01	< 0.01	2
No current use of corticosteroid (ref)	1				0
Current use of corticosteroid	3.60	1.99	6.51	< 0.01	2
Negative markers of inflammation (ref)	1				0
Positive markers of inflammation	2.31	1.15	4.66	0.02	1

*ICA*: ileo-colonic anastomosis, *IRR*: incidence rate ratio

IRR and score estimates were obtained by Poisson models based on clinical assessment findings; models evaluated the occurrence of each specified outcome within the subsequent clinical assessment; 95% confidence intervals and *p*-values were calculated taking into account that clinical assessments were clustered within patient; selected predictors for each outcome were obtained starting from a multiple model including all the variables described in the method section and then excluding at each step that with the highest *p*-value > 0.15 (from log-likelihood ratio test). Final models include only predictors with a log-likelihood ratio test *p* < 0.15; IRR > 1 indicate an increased risk of having the specified outcome compared to the reference group

### Factors predictive of the need to start azathioprine

**Fig. 3:**
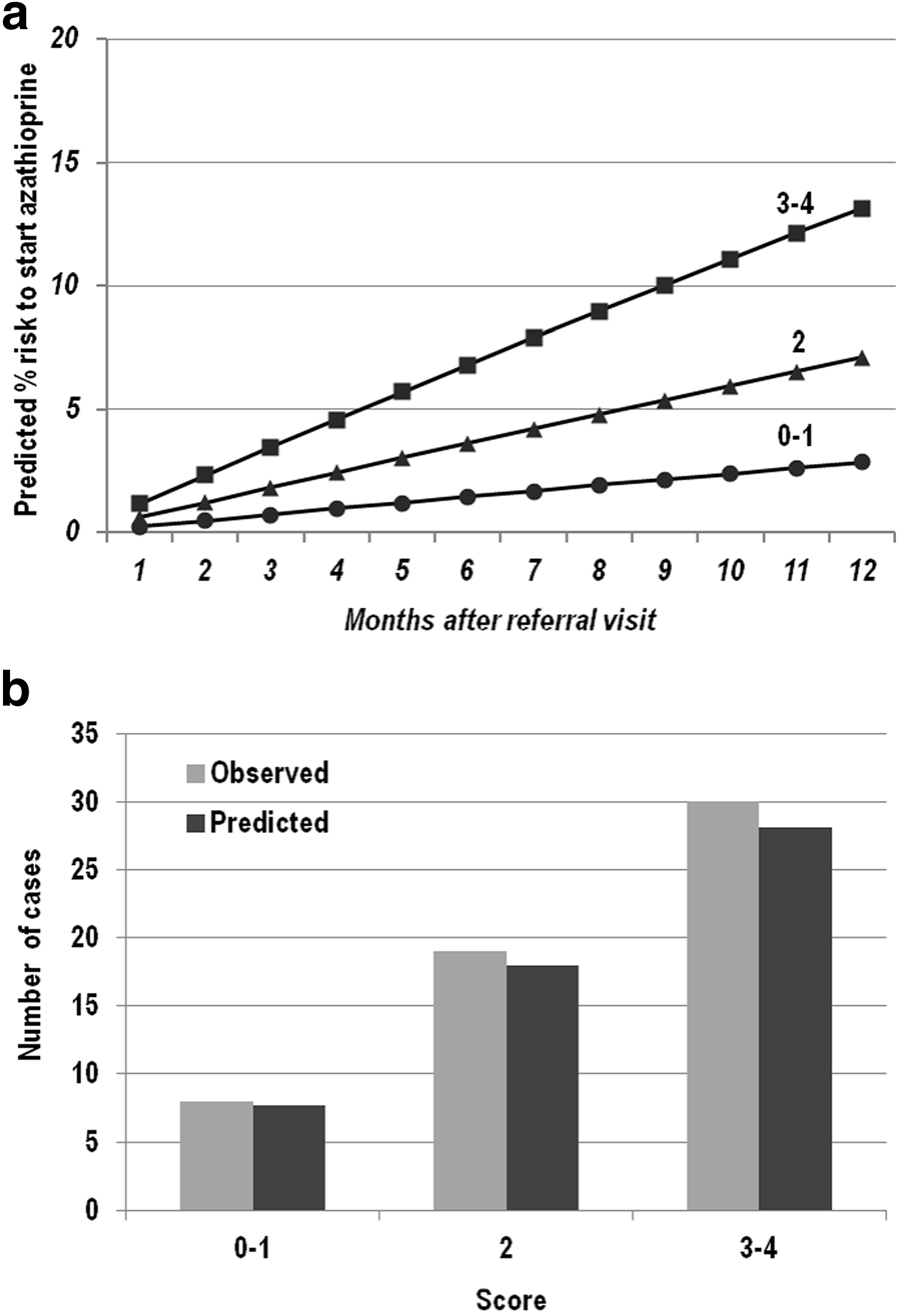
**a** Estimated cumulative probability of start azathioprine by month after referral visit for patients with different total score. **b** Observed vs model-predicted at one year since the referral visit of start azathioprine by groups of score

Sixty nine patients (43.1%) (45 female, median age at diagnosis 28.4 years, IQR 21.6–41.9) needed to start treatment with azathioprine. Three independent risk factors for the need to start azathioprine treatment within the next clinical assessment were identified (Table 2, section B), 1) female gender, 2) BMI value < 21, 3) CDAI > 50. The integer risk score ranged from 0 to 4 points and observations were grouped according to the following scoring categories, 0–1, 2 and 3–4, respectively. Figure 3a shows the predicted percentage risk to start azathioprine up to 12 months after the referral visit for patients within different score groups. Figure 3b compares observed and model-predicted start of azathioprine across the three risk groups according to the goodness-of-fit model.

### Factors predictive of the need to start anti-TNF-α drugs

**Fig. 4:**
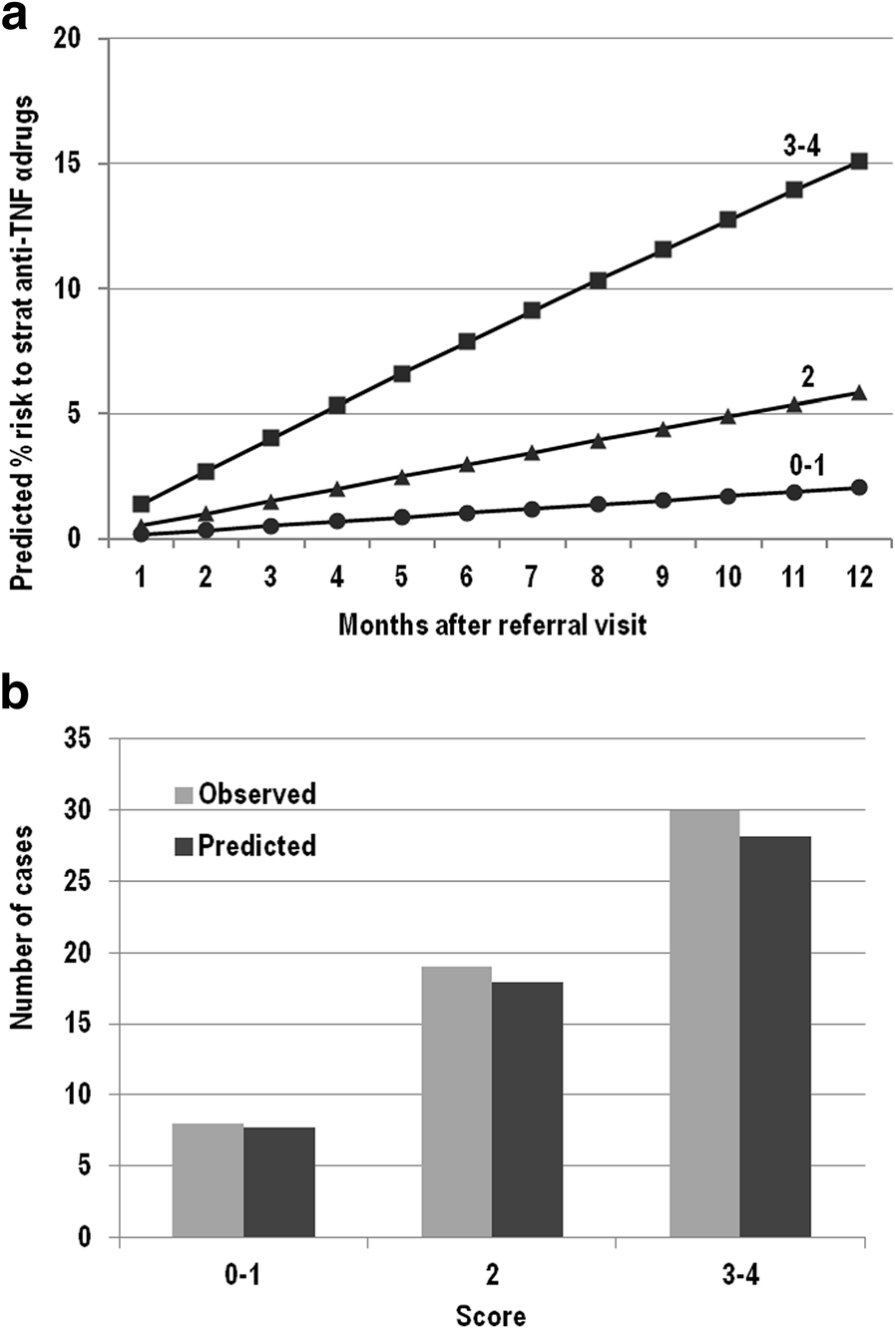
**a** Estimated cumulative probability of start anti-TNF α drugs by month after referral visit for patients with different total score. **b** Observed vs model-predicted at one year since the referral visit of start anti-TNF- α drugs by groups of score

Fifty-seven patients (35.6%) (33 female, median age at diagnosis 29.4 years, IQR 22.5–40.3) needed to start anti-TNF-α drugs. Three independent factors for the need to start anti-TNF-α drugs treatment within the next clinical assessment were identified (Table 2 section C), at SICUS evaluation the presence of, 1) CD complications, and small bowel CD lesion > 20 cm in absence of CD complications, 2) presence of specific intestinal symptoms, and 3) positive inflammatory markers. The integer risk score ranged from 0 to 4 points and observations were grouped according to the following scoring categories: 0–1, 2 and 3–4, respectively. Figure 4a shows the predicted percentage risk to start anti-TNF-α drugs treatment up to 12 months after the referral visit for patients within different score groups. Figure 4b compares observed and model-predicted start of anti-TNF-α drugs across the three risk groups according to the goodness-of-fit model.

### Factors predictive of the need of surgery

**Fig. 5:**
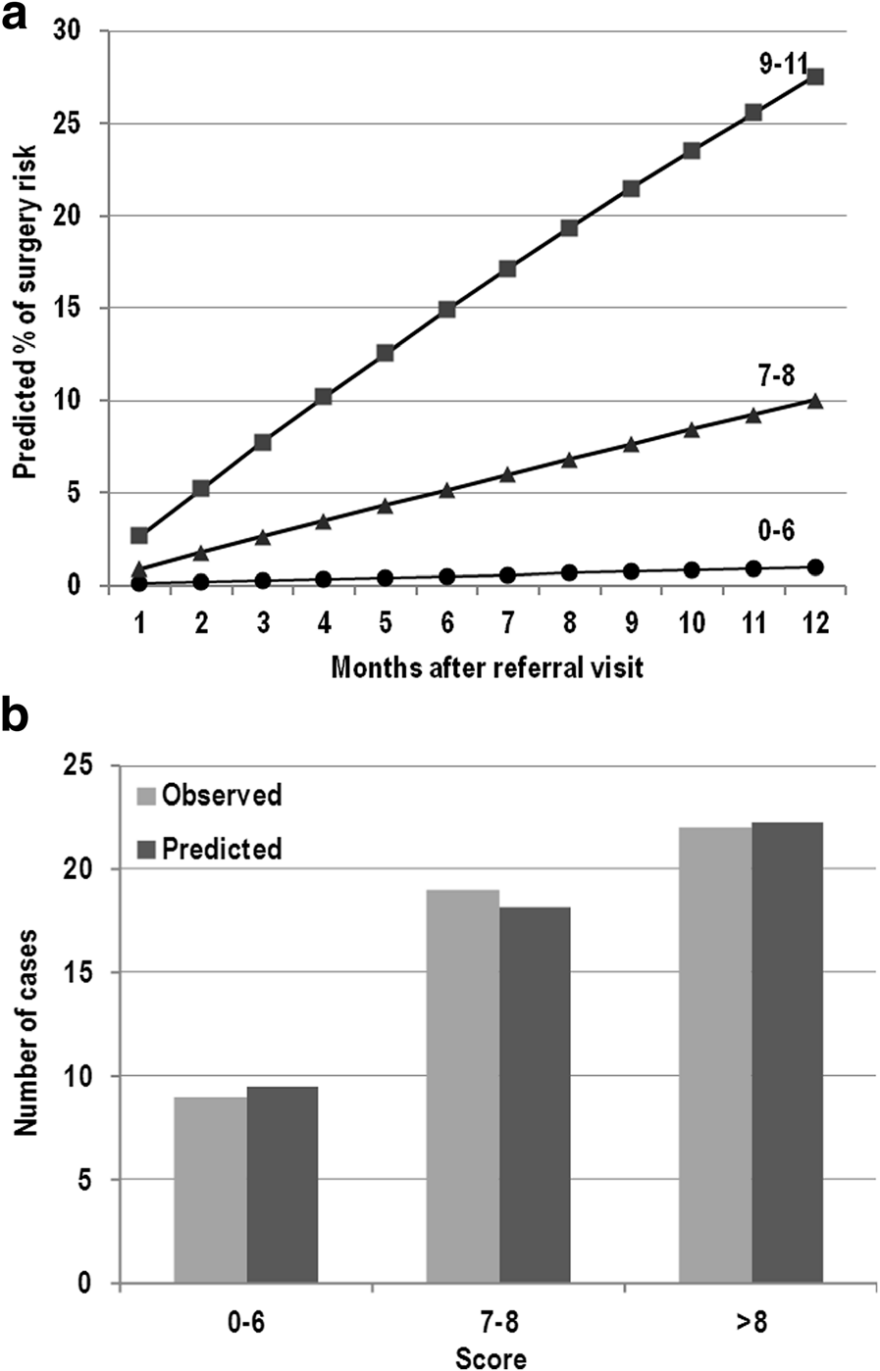
**a** Estimated cumulative probability of need of surgery by month after referral visit for patients with different total score. **b** Observed vs model-predicted at one year since the referral visit of need of surgery by groups of score

Fifty (31.2%) patients (28 female, median age at diagnosis 30 years, IQR 23.1–39.5), required surgical treatment on average 5 years from the diagnosis (IQR 1.6–9.9) and on average 16.1 months from the study enrolment (IQR 3.4–45.9). Six independent factors for the need of surgery within the next clinical assessment were identified (Table 2 section D): 1) the absence of previous surgery for CD or previous intestinal resection with termino-terminal ileo-colonic anastomosis; 2) the presence at SICUS evaluation of small bowel CD lesion length > 0.5 cm *plus* one or more complications; 3) the presence of specific intestinal symptoms; 4) no steroid requirement for treating the first flare-up of the disease; 5) the current use of corticosteroid; 6) positive inflammatory markers. The integer risk score ranged from 0 to 11 points and observations were grouped according to the following scoring categories: 0–6, 7–8 and 9–11, respectively. Figure 5a shows the predicted percentage risk of need of surgery up to 12 months after the referral visit for patients within different score groups. Figure 5b compares observed and model-predicted need of surgery across the three risk groups according to the goodness-of-fit model.

## Discussion

Surgery, corticosteroids, immune-suppressants and anti-TNF-α drugs are often required in CD patients, but a significant proportion of them requires less aggressive, or no, treatment (4). Reliable predictors of short and long-term patient outcome would allow to individually tailor therapy within a properly planned clinical follow-up. The outcome of any treatment of CD is determined by the clinical and pathological behavior and progression of the disease as well as by the response to treatment itself. However, in CD there are no unequivocal outcomes to assess the response to available treatments nor to quantify in a score model the predictive factors of severe disease. It is assumed that progressive bowel damage may, over time, result in the development of CD complications [1], nonetheless objective assessments of serial time-related disease modification and intestinal damage are lacking and it is not known whether the degree of bowel damage is an independent risk factor for disease progression. It has been recently shown that the Lémann index measures the cumulative bowel damage [16]. This index relies on high-quality abdominal MRI and radiology expertise, lacks, so far, of “gold standard” clinical references and is not applicable in clinical practice. To our knowledge, no prospectively estimated score indexes have been used to predict the CD clinical outcome, except the one proposed by Rutgeerts [17], based on endoscopic findings. In patients submitted to curative ileo-colon resection, SICUS is an accurate method for detecting early post-operative lesions and is comparable to the Rutgeerts score [12]. Differently from MRI, SICUS is based on a widely available technique not requiring costly and highly technological equipment and it has been proven to accurately assess CD small bowel intestinal lesions and complications both in adult and pediatric CD patients [6–9]. Of the 32 prospectively evaluated predictors, twelve independent predictors of the need of short-term treatment modification, including surgery, have been identified. In the present study, differently from previous studies assessing predictors of severe or disabling CD [2–4], the risk model has been converted into an integer score. This score can easily be translated in probability of the need of a short-term step-up therapeutic change. The most relevant result is that the predicted percentage of risk within 1 year was low across all the outcomes explored. In particular, the predicted risk of surgery varied from 1% (score 0–6) to 28% (score 9–11); that of starting azathioprine from 3% (score 0–1) to 13% (score 3–4); that of starting anti- TNF-α drugs from 2% (score 0–1) to 15% (score 3–4). These results are consistent with those reported by two studies, a large retrospective cohort [4] and a population-based study [18], showing that the probability of surgery and severity course of CD is overall low.

Bowel damage, as assessed by SICUS, is per se the most important predictor for any of the considered outcomes except for the need to start azathioprine. Age at diagnosis < 40 yrs is predictive for the use of corticosteroids and not for the other outcomes. A prior retrospective cohort study [2] found that younger age (< 40 yrs) at diagnosis was a predictive risk factor of disabling disease, alone or in association with steroids treatment for the first flare-up and presence at diagnosis of perianal disease. The different results of the present and Beaugerie et al. studies can be explained by the different short-term outcome (i.e., the need of therapeutic change or surgery within the next clinical assessment), the different number of predictors evaluated, 32 vs 9, and the exclusion in the present study of patients with childhood CD onset. Similarly to previous studies, we did not find an association between the short-term need of surgery and the use of azathioprine and anti-TNF-α drugs [19–23]. The association between the current use of corticosteroids and the need of surgery confirms the results of the Danish nationwide population-based cohort study [24]. The absence of previous surgery was associated with the need of surgery indicating that surgical removal of diseased bowel is followed by a favorable short-term clinical outcome [25]. We did not find an association between the need of surgery and the early age of onset and long duration of the disease [26–28]. The analysis here performed was adjusted for the age at diagnosis and at enrollment, and for the duration of follow-up. At diagnosis more than two third of patients had stricturing and penetrating disease and one third perianal disease, ruling out an inclusion bias. Confirming a previous retrospective study [29], complicated disease at diagnosis had no predictive value for the need of surgery or step-up therapy except for the association of stricturing disease with the use of corticosteroids. The duration of the disease, the time interval between diagnosis and enrollment were not associated with the need to start azathioprine or anti-TFN-α drugs. The analyses were adjusted for the use of any other treatment. Female gender, CDAI > 50, and a low BMI concur in a score that predicts the start of azathioprine.

CD lesions of the upper gastrointestinal (GI) tract have been variably reported (28) to be associated with younger age at diagnosis, stricturing disease and two or more abdominal surgeries. Due to the small number of patients with upper CD, as expected in adult CD patients, we did not evaluate separately the upper CD location as a predictive risk factor for any of the outcomes evaluated.

At multivariate analysis neither the extension of intestinal lesions at SICUS nor the length of intestine resected at surgery, adjusting for all potential confounding variables including immunosuppressive therapy and smoking habit, had an independent predictive effect on the need of surgical outcome. Conversely, the extension of the intestinal lesions evaluated at SICUS was independently associated with the short-term outcome to use steroids and to start anti-TNF-α drugs.

Similarly to population-based cohort and tertiary referral studies, we did not find an independent association between smoking status [29–32] and family history [33, 34] (defined on subject report) for IBD and the need of surgery or step-up treatment. The presence of family history for IBD was accurately assessed enquiring the patients repeatedly at each clinical assessment therefore updating the new diagnoses occurring among relatives during the follow-up. The smoking behavior and the current number of cigarettes per day were assessed at each clinical assessment according to the threshold value previously published, avoiding recall bias [35].

Some drawbacks of the study need to be mentioned. This is a cohort study evaluating CD patients afferent to tertiary referral centers for IBD so they could not be representative of the patients population seen in a general gastroenterological setting. It is of note that, in our population at enrollment, one third of patients were previously submitted to one or more surgeries, one third had perianal disease and more than half had CD complications while patients with CD of the colon were a minority. The study was based on only 160 patients. As expressed by the 95% CIs estimated, for some predictors identified, the estimated effect on the evaluated outcomes presents a relatively large uncertainty even if the analysis was performed on 1464 assessments, thus increasing the statistical power [36]. Furthermore, due to the limited number of patients, we had to group some categories thus missing the possibility of detecting differences within those categories. Inflammatory markers (ESR and CRP) were considered as dichotomy variables not evaluating different cut-off value [37]. Lastly, we should consider that SICUS, as all ultrasound methods, is operator-dependent. It should be pointed out, however, that the score described in this study may be applied with any technique, including MRI, that allows to accurately assess the length of intestinal lesions and occurrence of CD complications.

The strength of the study is that patients were invited to outpatient clinic at regular time intervals, regardless of symptoms, thus minimizing the influence of follow-up duration in the development of severe course of CD and including in the analysis also asymptomatic patients. In addition, we enrolled in the study patients from 2002 when anti-TFN-α drugs became available and widely used in the hospitals of Lazio region, Italy. Thus the availability of drug treatments did not affect the proportion of patients that started azathioprine and anti-TNF-α drugs.

Differently from previous studies that rely mainly on data assessed retrospectively on clinical records or on mailed questionnaires, in this study at each clinical assessment all potential predictors of having, within the next clinical assessment, one of the considered outcomes were evaluated prospectively in a systematic way. Although the outcomes were arbitrarily chosen based on available therapeutic options and chosen on clinical grounds, it is of note that these choices were done by gastroenterologists with over 20 years experience in IBD in accordance with the diagnostic classification and treatment protocols of the ECCO consensus guidelines. The scores here proposed were based on the selection of some predictors by goodness of fit among 32 variables initially considered. Each predictor included contributes to the total score with a different weight based on the ability of that characteristic of predicting the outcome considered independently from all other predictors included. By assessing clinical factors, usually taken into consideration in the gastroenterological practice, this study has identified the main risk factors that make up four scores able to predict the four most relevant short-term clinical treatment strategy changes in the management of CD patients.

## Conclusion

The four risk scores developed here, should be submitted to a future validation in a larger population and possibly in several centers. In conclusion, the identified scores provide a useful tool for clinicians allowing an objective prognostic assessment of individual patients with Crohn’s disease, including an estimation of the need for treatment adjustment. Such timely awareness of the patient risk profile may be of value for physicians in determining the most appropriate management and treatment of patients with Crohn’s disease.

## Abbreviations

B1, B2, B3: Behavior 1,2,3
BMI: Body mass index
CD: Crohn’s disease
CDAI: Crohn’s disease activity index
CRP: C reactive protein
ESR: Erythrocyte sedimentation rate
IBD: Inflammatory bowel disease
ICA: Ileo-colonic anastomosis
IQR: Interquartile range
IRR: Incidence rate ratio
L1,L2,L3,L4: Location,1,2,3,4
MRI: Magnetic resonance imaging
PD: Perianal disease
SICUS: Small intestine contrast ultrasonography
TNF-α: Tumor necrosis factor-α
